# Fetus In Fetu — A Mystery in Medicine

**DOI:** 10.1100/tsw.2007.56

**Published:** 2007-02-19

**Authors:** A. K. Majhi, K. Saha, M. Karmakar, K. Sinha Karmakar, A. Sen, S. Das

**Affiliations:** Department of Obstetrics and Gynecology, NRS Medical College, Kolkata 700014, West Bengal, India

**Keywords:** fetus-in-fetu, monozygotic diamnionic twinning, parasitic twin, teratoma

## Abstract

Fetus in Fetu (FIF) is a rare condition where a monozygotic diamnionic parasitic twin is incorporated into the body of its fellow twin and grows inside it. FIF is differentiated from teratoma by the presence of vertebral column. An eight year old girl presented with an abdominal swelling which by X-ray, ultrasonography and CT scan revealed a fetiform mass containing long bones and vertebral bodies surrounded by soft tissue situated on right lumber region. On laparotomy, a retroperitoneal mass resembling a fetus of 585 gm was removed. It had a trunk and four limbs with fingers and toes, umbilical stump, intestinal loops and abundant scalp hairs but was devoid of brain and heart. Histology showed various well-differentiated tissues in respective sites. FIF is a mystery in reproduction and it is scarce in literature in such well-developed stage.

## INTRODUCTION

Fetus in fetu (FIF) is a rare condition where a monozygotic diamnionic, parasitic twin is incorporated into the body of its fellow twin early in embryonic development and grows inside it through a vascular communication with the host circulation.

The term fetus-in-fetu (FIF) was coined by Meckel in 18$^{th}$ century and defined by Willis[1] in 1953 as a mass containing a vertebral axis often with appropriate arrangement of other organs or limbs around this axis by which it is differentiated from the highly differentiated teratoma.

## CASE REPORT

An 8-year-old girl was admitted with a complaint of upper abdominal discomfort and gradually increasing swelling of the upper abdomen for last two years. Her mother had another healthy male child of 4 years. Both of them were delivered by lower uterine cesarean section. Her mother had also one spontaneous abortion and one stillborn delivery. Both the parents were in good health. There was no history of twinning in the family.

On examination the height of the girl was 3 feet 10 inches and weight 16 kg. There was a lump on right hypochondrium encroaching on to the epigastric, right lumber and umbilical regions, measuring about 15cm × 12cm with lobulated surface, firm consistency, and restricted mobility without any tenderness.

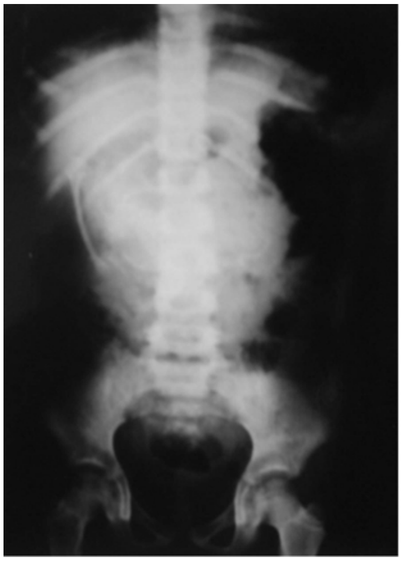

**FIGURE 1.** X-ray shows areas of calcification and long bone in right upper abdomen.

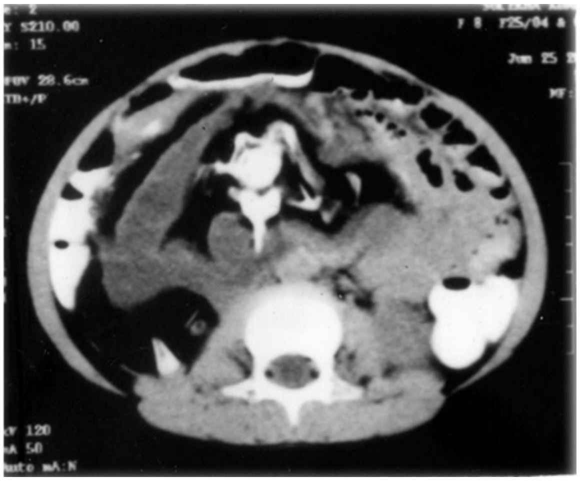

**FIGURE 2.** Transverse CT scan of upper abdomen of the girl showing soft tissue, fat and bony elements including vertebral body and long bone.

Ultrasonography showed a large echogenic mass with heterogeneous echo texture with some cystic spaces and areas of calcification. Liver function test and serum alpha-fetoprotein level were within normal limits. The plain film (Fig. 1) of the abdomen showed long bone looking like fetal femur situated on the upper abdomen obliquely extending from first to third lumbar vertebrae of the child. There was another calcified mass resembling vertebral column. An abdominal computed tomography (CT) scan (Fig. 2) showed complex intra-abdominal mass (18.5cm × 15cm) showing bony components identical to fetal femur and spine with soft tissue surrounded by fat density mass representing fetus-in-fetu. Bowel loops were displaced by this mass. Based on imaging findings a diagnosis of fetus in fetu was made and decision of laparotomy was taken.

On laparotomy, through upper abdominal transverse incision, a huge retroperitoneal encapsulated mass resembling a fetus was detected behind the transverse mesocolon. It had a transverse disposition with a femur lying on the right side and the incomplete cranium lying in front of and left to the vertebral column of the child. The mass appeared to get vascular supply from the branches of pancreaticoduodenal artery. It was dissected from the surrounding structures like duodenum, common bile duct, pancreas, right kidney and ureter with difficulty. On its cranial aspect there were abundant hairs. Uterus and adnexae of the girl were small but normal. The mass was removed intact (Fig. 3). Postoperative period was uneventful and the girl was discharged after two weeks. On follow up after six months she was found well with normal sonographic findings.

On gross examination, the mass was a fleshy structure resembling a fetus (Fig. 3, 4) measuring 15 cm × 20 cm and weighing 585 gm. It had a trunk with four limbs. The lower limbs were well developed containing four toes on each foot with large nails. Both upper limbs were ill developed with left one more rudimentary. At the cranial part there was no flat cranial bones, there was only a small base of the skull covered by a scalp with abundant hair. The trunk and the limbs were covered with peeled off skin. The chest portion was almost solid. On the abdominal region there was an umbilical stump and on perineal region there were two dimples situated anteroposteriorly on the ventral aspect but sex could not be differentiated. On dissection of the ventral aspect (Fig. 4) a small peritoneal cavity containing small loops of intestine of approximately 5 mm of diameter was found (Fig. 4). The intestine ended on a blind organ probably undifferentiated cloacae. On the posterior wall of the cavity hard vertebral column was clearly visible but heart, lung, diaphragm, liver and spleen could not be identified separately. Placenta or placental tissue could not be identified.

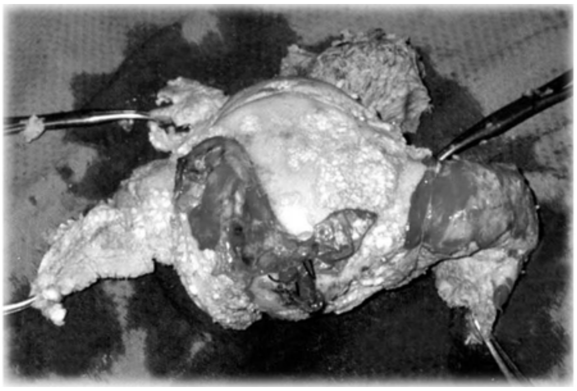

**FIGURE 3.** Showing the specimen (fetus-in-fetu) in dorsal view, forceps pointing to four limbs

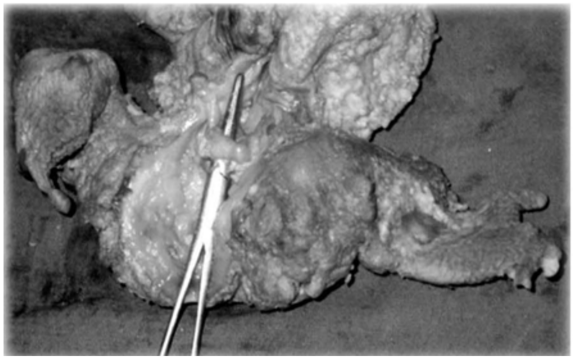

**FIGURE 4.** Showing ventral aspect of fetus-in-fetu, forceps showing intestinal loop

X-ray of the specimen (Fig.5) showed vertebral column, base of the skull and long bones.

Histological study revealed presence of skin, muscle, nerve tissue, cartilages, intestines, blood vessels, bones and dystrophic calcification. Umbilical stump had two vascular lumen. Karyotyping was not done.

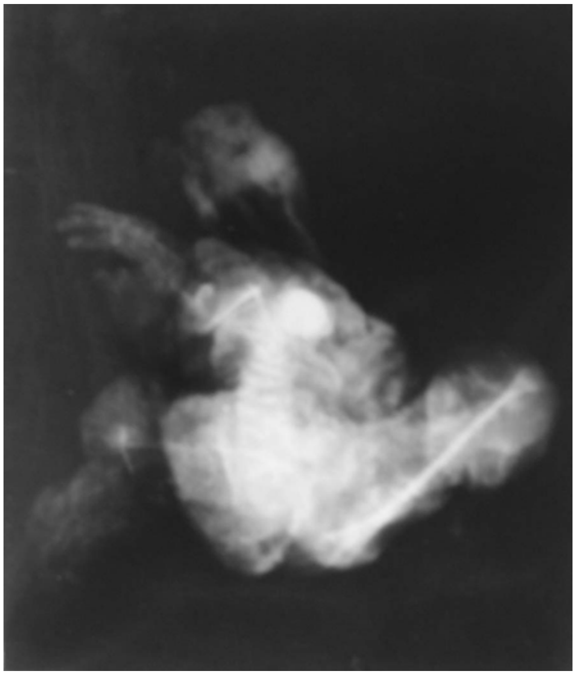

**FIGURE 5.** X ray of the specimen (fetus-in-fetu) showing vertebral column, ill formed base of the skull and limbs including a large femur.

## DISCUSSION

The incidence of FIF is 1 in 500,000 births[2] and to date fewer than 100 cases have been reported in the world literature. It is a variety of monozygotic diamnionic twinning resulting from unequal division of the totipotent inner cell mass of the developing blastocyst leading to the incorporation of a smaller cell mass within a maturing sister embryo. Initially rate of growth of FIF parallels with fellow twin but suddenly the growth of FIF stops due to inadequate blood supply or inherent defect. The blood supply of a FIF commonly comes from the abdominal wall plexus. However, superior mesenteric artery, inferior mesenteric artery or renal artery may also supply[3]. In a recent review of 87 cases done by Hoeffel et al.[3] various information on this subject have been highlighted. Most of the reported cases are in newborns and infants. In 89% of cases, FIF was detected before 18 months of age, the highest age reported is 47 years; there is a slight preponderance in boys than girls. In 88% of the cases a single parasitic fetus is found, but multiple fetuses as high as five have been reported. The weight ranged from 1.2 gm to 1.8 kg with size varying from 4cm to 24.5cm. FIF is found to be located mostly (80%) in retroperitoneal area majority being in upper abdomen, but also have been found in unusual locations like skull, sacrum, adrenal gland, mouth, lungs, and even in the scrotum. FIF is almost always incompletely developed. The vertebral column and limbs are found to be present in almost all cases (91% and 82% respectively). The lower limbs are more developed than the upper limbs. Central nervous system, gastrointestinal tract, vessels and genitourinary tract are found present in 55.8%, 45%, 40% and 26.5% respectively. Presences of adrenal gland, respiratory tissue, splenic tissue, lymphoid tissue, and pancreatic tissue in FIF have also been reported[4]. Umbilical cord is composed usually of two vessels. FIF are always anencephalic[3] and presence of heart is rarely found[4]. When gonads are found, histologically they are of the same sex as that of the host[5]. Karyotype of FIF is similar to that of the host[6].

The most common presentation (70%) in FIF is an asymptomatic abdominal mass[7]. Sometimes there may be abdominal discomfort with or without distension, feeding difficulties, vomiting, jaundice, and dyspepsia. Symptoms may appear due to rapid growth and pressure effects on adjacent organs. Signs of maceration with the threat of consumptive coagulopathy had been reported by some authors[8]. FIF may hinder descent of testes in a male infant[9]. FIF may be incidentally discovered during ultrasonography. Abdominal mass found in prenatal period on routine obstetric sonography has been diagnosed to be a case of FIF following delivery and laparotomy during neonatal period[4,9]. The diagnosis is based on the findings of abdominal radiography and ultrasonography. Before 1990, less than 20% cases were diagnosed pre-operatively. Availability of CT-scan has facilitated the pre-operative diagnosis. A mass predominately consisting of fat or encapsulated fluid that surrounds a central bony structure is a typical finding of FIF by CT. Recently MRI is being used to diagnose it. CT is not only helpful for diagnosis but it also evaluates the extent and the vascular supply, the knowledge of which is necessary for accurate surgical dissection.

Differential diagnosis of FIF is teratoma and meconium pseudo cyst. Though FIF and teratoma are considered to be the part of a spectrum of the same disorder[10], they are, for obvious reasons, thought to be the separate pathological entities. The characteristic distinguishing features of FIF from teratoma are the presence of vertebral column and very high degree of development of organs and limbs[1]. The presence of vertebral column suggests that the parasitic fetus has developed at least to the primitive streak age, (12 to 15 days gestation age) to develop a notochord from which the vertebral column is developed[11]. The spinal column cannot be identified sometimes by images because it may be relatively radiolucent or markedly dysplastic. Gonzalez-Crussi[12] defined FIF as “any structure in which the fetal form is in a very high development of organogenesis and to the presence of a vertebral axis”. In contrast, teratoma is the accumulation of pluripotent cells without any organogenesis or without presence of vertebral segmentation[13]. Genetic identity between the host and fetiform mass using genetic markers of chromosomes is found in FIF but not in malignant teratoma[14].

Complete resection is the treatment of choice of FIF and is usually curative. Sometimes complete excision is not possible due to the adherence to the host‘s organs. Case has been reported to manage conservatively without surgical intervention[10]. Although it is considered to be benign in nature, malignant transformation is not unknown[15]. Prognosis for FIF is more favorable than teratoma. Close clinical, radiological and serological follow up is needed due to the presence of immature elements[15].

## CONCLUSION

Fetus-in-fetu is a rare but a wonder in medicine. With the help of current imaging modalities nowadays, it has been easier to diagnose FIF prior to surgery and even during prenatal periods. With multidisciplinary approach management becomes easier and complete excision is curative.

## ACKNOWLEDGEMENT

We gratefully acknowledge the contribution of Professor Basanta Kumar Khan, Medical Superintendent cum Vice Principal; Professor Sudipta Bose, Head of the department, G&O; Dr Hiralal Konar, Medical Officer, Pediatric Surgery, NRS Medical College, Kolkata for publishing this work.
